# Mother–Preterm Infant Contingent Interactions During Supported Infant-Directed Singing in the NICU—A Feasibility Study

**DOI:** 10.3390/children12091273

**Published:** 2025-09-22

**Authors:** Shulamit Epstein, Shmuel Arnon, Gabriela Markova, Trinh Nguyen, Stefanie Hoehl, Liat Eitan, Sofia Bauer-Rusek, Dana Yakobson, Christian Gold

**Affiliations:** 1 School for Creative Arts Therapies, University of Haifa, Haifa 3498838, Israel; sepstein3@staff.haifa.ac.il; 2 Faculty of Psychology, Department of Developmental and Educational Psychology, University of Vienna, 1010 Vienna, Austria; trinh.nguyen@iit.it (T.N.); stefanie.hoehl@univie.ac.at (S.H.); 3 Gray Faculty of Medical and Health Sciences, Tel Aviv University, Tel Aviv 69978, Israel; 4 Neonatal Department, Meir Medical Center, Kfar Saba 4428132, Israel; 5 Institute for Early Life Care, Paracelsus Medical University, 5020 Salzburg, Austria; gabriela.markova@pmu.ac.at; 6 Neuroscience of Perception and Action Lab, Italian Institute of Technology, 00161 Rome, Italy; 7 Department of Developmental and Biological Psychology, University of Heidelberg, 69117 Heidelberg, Germany; 8 Physical Therapy Unit, Meir Medical Center, Kfar Saba 4428132, Israel; 9 NORCE Research AS, Postboks 22 Nygårdstangen, 5838 Bergen, Norway; chgo@norceresearch.no; 10 Department of Clinical and Health Psychology, University of Vienna, 1010 Vienna, Austria

**Keywords:** preterm infants, infant-directed singing, contingency, parent–infant interactions

## Abstract

**Highlights:**

**What are the main findings?**

- We demonstrate feasible behavioral scales to measure contingency between mothers and preterm infants’ behaviors during supported infant-directed singing.
- Preterm infants exhibit regulated behaviors during supported mother-led, infant-directed singing.

**What are the implications of the main findings?**

- Supported infant-directed singing may facilitate regulation.
- This regulation of infant-directed singing may benefit developmental outcomes of preterm infants.

**Abstract:**

Background: Supported infant-directed singing (IDS) for parents and their preterm infants has proven beneficial for parents and preterm infants’ health and relationship building. Studying parent–infant contingent interactions through behavioral observations is an established method for assessing the quality of interactions. Very few studies have measured contingency between parent and preterm infants in the neonatal period during supported IDS. Methods: We conducted a feasibility study to assess the possibility of analyzing parent–very preterm infant dyads’ contingency during supported IDS in the NICU. We recruited four mother–infant dyads and video-recorded a single music therapy (MT) session before their discharge from the hospital. Two independent researchers coded three selected segments (beginning, middle, and end) from each video, according to adapted behavioral scales with inter-rater agreement analysis. Contingency between infant and maternal behaviors was analyzed. Results: Twelve video segments were coded. High inter-rater agreements (Cohen’s kappa) were found for infant eye-opening (0.93), hand positions (0.79), and head orientation (0.94), as well as maternal head orientation (0.95) and vocalizations (0.95). During supported IDS, increased infant head orientation toward the mother, eyes closed, as well as maternal head orientation toward the infant (all *p* < 0.001), were recorded compared to no IDS. Direction of the maternal head toward her infant was contingent on the infant’s closed eyes, extended hands, and head not toward mother. Conclusions: This feasibility study demonstrates contingency between mothers and their preterm infants’ specific behaviors during IDS. These interactions can be analyzed through video segments with high inter-rater agreement. The method described might help in evaluating other modalities that might be related to contingency. Recent advances in AI can make this tool easier to accomplish, with further studies to evaluate the importance of contingency for child development. The findings suggest that supported IDS influences infant attention and regulation.

## 1. Introduction

Preterm birth is a global health concern with short- and long-term consequences for both infants and their families [1,2,3,4,5]. Preterm infants, born before 32 weeks of gestation, have higher morbidity rates and a greater likelihood of brain injury. As a result, they are considered at risk for neurodevelopmental impairments [6,7,8]. During their hospitalization period, preterm infants spend less time in alert states, are less responsive in social interactions, and send less clear communicative signals compared to infants born at term [9,10,11,12]. Parents of preterm infants exhibit less responsive and attuned responses to their infants [13,14,15]. As a result, interactions between caregivers and preterm infants tend to be less mutually responsive and adaptive compared to those of full-term infants [10,16,17,18]. Infants learn and develop through interactions with others, initially with their primary caregivers. Fostering interactions and emotional bonds between mothers and their preterm infants is therefore critical to enhancing the family’s well-being and an infant’s healthy development [19,20].

One promising intervention to enhance these interactions in the NICU is supported infant-directed singing (IDS), which has shown potential in fostering parent–infant bonding (IDS) [21,22]. The use of IDS in the neonatal context is based on a large theoretical and empirical literature detailing the musical nature and components of parent–infant early interactions and their benefits for infant development [23,24,25,26,27]. When entrained and attuned to the infant’s biological and behavioral states, supported IDS provides essential auditory stimulation for the preterm infant, fostering both emotional bonding and physical closeness between parent and infant [22,28]. Supported parent-led IDS is a common intervention in neonatal music therapy (MT) with short-term beneficial outcomes documented in several systematic reviews [29,30,31,32]. One recent study measured the long-term effects of MT on parental bonding and infant development, showing positive experiences reported by caregivers [33] but no effects on mother–infant bonding [34].

To better understand the effects of supported IDS, it is essential to examine the processes occurring during IDS and how parents and their preterm infants coordinate their interactions. Parent–infant interactions have been extensively studied, with research linking these interactions to long-term neurodevelopmental outcomes [10,35,36,37,38,39]. One crucial process emerging from the research is contingency, which refers to the mutual association and co-occurrence of behaviors between the parent and infant [18,40,41,42,43,44]. Both young and preterm infants are capable of detecting contingency in spontaneous interactions [18,41,45,46]. In addition, preterm infants coordinate their vocalizations to the temporal aspects of maternal IDS [47,48].

Despite these findings, there remains a significant knowledge gap regarding the processes that occur during supported IDS in the NICU and how they might contribute to relationship-building of the parent and infant. Additionally, while contingency across different behavioral modalities is recognized as an important factor in infant development, it has not yet been systematically studied within the context of IDS in the NICU [44]. Given the defined ability of preterm infants to communicate, contingency seems particularly relevant to observe in parent–preterm infant interactions in the IDS context.

### Aim

The present study aimed to determine the feasibility of conducting a quantitative microanalysis of coordinated interactions between preterm infants and their mothers during supported IDS. To achieve this goal, we aimed to develop operational definitions of infant and maternal behaviors that can be assessed in this population and to achieve high inter-rater reliability; examine whether these observed behaviors change throughout an IDS session; and identify behaviors that may be suitable for measuring contingency. We hypothesized that contingency and/or synchrony would be observed in the behaviors of mothers and their preterm infants during IDS.

## 2. Materials and Methods

We conducted a pilot study with four mother–infant dyads (all stable very preterm infants) to evaluate the feasibility of assessing contingency during supported IDS in the neonatal intensive care unit (NICU). We planned to use time-series analysis such as cross-recurrence quantification analysis (CRQA) [49], to calculate parent–infant synchrony/contingency; however, the infants in our study were mostly asleep and showed a low frequency of variations in their behaviors, which are necessary for using time-lagged analyses [18,50,51]. We therefore abstained from using known time-series models and focused on finding associations between parental and infant behaviors.

### 2.1. Participants

We recruited four mother–preterm infant dyads using pragmatic sampling at the Meir Medical Center, Kfar Saba, Israel. The inclusion criteria were (1) preterm infants born at less than 31 + 6 weeks of gestation, (2) 33–37 weeks of post-menstrual age (PMA) and still in the NICU, (3) medically stable, and (4) had participated in supported IDS during MT as a part of their NICU hospitalization, to ensure their familiarity with the intervention during video recording. We excluded parents with insufficient understanding of Hebrew or with a known mental illness or cognitive impairment. Table 1 presents participants’ characteristics, including major neonatal morbidity such as intraventricular hemorrhage (IVH), categorized into 4 grades according to the Papile classification [52]; periventricular leukomalacia (PVL); bronchopulmonary dysplasia (BPD), classified as the need for oxygen after 36 weeks corrected age, the duration of oxygen support for respiratory stabilization; and retinopathy of prematurity (ROP) as diagnosed before discharge.

### 2.2. Intervention and Procedure

We conducted and recorded a single supported IDS session with each dyad before their hospital discharge. Two video cameras and one audio recorder were placed in front of the mother and above the mother’s shoulder to capture the maternal face and upper body, the infant’s whole body and face, and maternal vocalizations. Supported IDS followed a resource-oriented, family-centered, culturally sensitive, and individualized approach. Two certified music therapists delivered the intervention, which aimed to support parents in singing to their infants in a way that is attuned to infants’ behavioral states and developmental needs, incorporating the family’s musical preferences [53,54]. The beginning of each session consisted of relaxation with an ocean disc that lasted a few minutes. This was followed by a singing section, which lasted for about 20 min and consisted of singing songs-of-kin (family’s chosen songs adapted into lullaby style [55]), or lullabies, individually selected by the mothers, with occasional improvised lyrics. To match the infant’s developmental stage, the music therapist supported the mother in gradually introducing the singing section, starting with quiet humming that evolved into singing with lyrics.

We selected three musical parts from each video to enable a comparison between the beginning, middle, and end of sessions as a process-oriented analysis. The selected segments were as follows: the first 2.5 min (relaxation with ocean disc); 2.5 min from when the first song started; and the last 2.5 min of the session (consisting of the last song). This resulted in a total of twelve 2.5 min videos across all participants (twelve from mothers’ camera and twelve from infants’ camera).

### 2.3. Development of Behavioral Coding

We micro-coded the following behaviors:

- Maternal behaviors: affect; head orientation; vocalizations (second-by-second, i.e., 1 rating per s); movements of hands, head, and legs (frame-by-frame, i.e., 1 rating per 1/30 s).
- Infant behaviors: head orientation; eye-opening; hand positioning (second-by-second); movements (frame-by-frame).

The behaviors were operationalized through numerical scales representing the exact behavior (Table 2). The operational definitions were adapted from previous studies with term and preterm infants [18,51,56,57].

In addition to the micro-level codification, mother–infant interactions were globally rated using macro scales (150 s, i.e., 1 rating for the whole video):

- Maternal behaviors: overall affective tone.
- Infant behaviors: body orientation; state of alertness [58].

Two trained assessors (SE, LE) conducted the coding using ELAN 6.7 coding software. We selected the video segments of dyad D for coding training and refinement. This dyad was selected due to the comparatively active state of the infant, providing more infant behaviors to observe and code. This dyad was used as a learning case only and therefore not included in the inter-reliability analysis as well as in the statistical analysis. During this first stage of the microanalysis, the coders performed the analysis together and discussed any disagreements until a complete agreement occurred. This led to a refinement of the scales to achieve acceptable reliability. Videos of the remaining dyads (A–C) were coded by both raters independently, and inter-rater reliability was calculated using the built-in functions in ELAN. Some behaviors posed challenges in reaching high inter-rater reliability, leading to a second refinement of the scales and a second coding round.

### 2.4. Statistical Analysis

Inter-rater reliability: During the training and refinement process, we used Elan 6.7 software to calculate Cohen’s kappa (iterative proportional fitting, IPF, algorithm [59]) separately for each video. In the final data, we used RStudio version 2024.0402+764 for all numeric and graphical analyses, with R package psych to calculate Cohen’s kappa with 95% confidence intervals (CIs), combining data across all videos. 

Graphical analysis of micro behaviors: To understand patterns in the data, we analyzed all coded behaviors graphically.

Contingent interaction calculations: Fisher’s exact test was used to examine contingency between behaviors.

### 2.5. Ethics

The research protocol was approved by the responsible institutional review board and the Meir Medical Center in Israel, approval no. 0276-23-MMC, 18 January 2024. Eligible mothers were asked to sign informed consent, ensuring their confidentiality, privacy, and right to withdraw. Recruitment was performed by one of the research team members, a physician in the NICU, who was not involved in delivery of supported IDS.

## 3. Results

### 3.1. Description of Observed Behaviors

#### 3.1.1. Macro Behaviors Are Shown in Supplementary Table S1

Infant body orientation was frontal in 11 of 12 segments.

Infant state of alertness: In all segments, the infant was either asleep (six segments) or drowsy (six segments).

Maternal affective tone was neutral in most segments (6) and positive in the mother D (all segments) and mother C end segments. Negative overall affective tone was seen in mother A (beginning and end segments) for one rater only.

#### 3.1.2. Micro Behaviors Are Shown in Figure 1 (Dyads A–C) and Figure 2 (Dyad D)

We separated the figures of dyads A, B, and C from D since dyad D was used as a learning case; therefore, inter-rater agreement rates were not calculated for this dyad. In addition, frame-by-frame coding (1 rating per 1/30 s) was performed only for dyad D, and it is presented in Figure 2.

**Figure 1:**
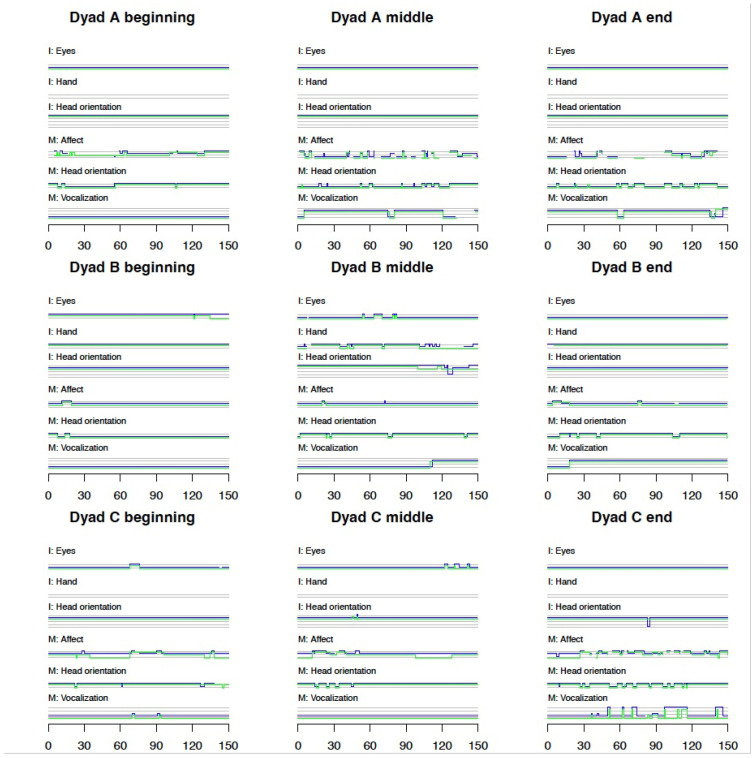
Overview of coded behaviors over time (dyads A–C). Note. Blue—rater 1; green—rater 2; grey-ticks of the behavioral scale. Behaviors I—infant behaviors; M—maternal behaviors. For each behavior, the ticks represent the behavioral scale, as described in Table 2. For example, maternal vocalization: highest tick—adult speech, second tick—infant-directed singing, third tick—infant-directed speech, lowest tick—no vocalizations.

**Figure 2:**
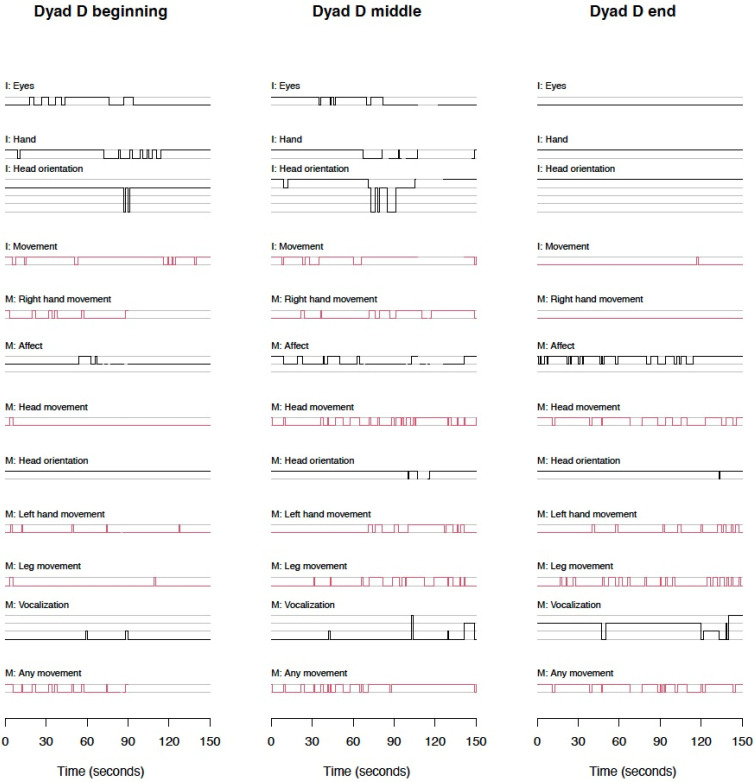
Overview of coded behaviors over time (dyad D). Note. Red—1/30 s scale; black—1 s scale. Behaviors: I—infant behaviors; M—maternal behaviors.

Given that infants were asleep or drowsy, infant eyes (I: Eyes) showed no or little variation and they were closed most of the time.

Infants’ hands (I: Hand) were covered and therefore not codable (grey lines) in six video segments (infants A and C). Variations between “centered” hands and “extended” hands were seen in three segments (infant B, middle; infant D, beginning and middle).

Infants’ head orientation (I: Head orientation) was usually toward mothers (highest tick) or frontal (2nd tick). Variation between head frontal (2nd tick), head toward mother (highest tick), and head away from mother (lowest tick) was seen in infant B, segment 2 and infant D, segment 2.

Maternal affect (M: Affect) Variation between positive affect (highest tick) and negative affect (lowest tick) was seen in infant A, all segments. Variation between positive affect (highest tick), neutral (middle tick), and negative affect (lowest tick) was seen in infant C, all segments. Several segments showed frequent variations (infant A, all segments; infant C, end; infant D, middle, end).

Maternal head orientation (M: Head orientation) was usually toward infant. Several segments showed frequent variations (infant A, middle, end; infant C, end).

Maternal vocalization (M: Vocalization): At the beginning of segments in all infants (relaxation with ocean disc), there were almost no vocalizations (lowest tick). Singing (2nd tick) was seen in six segments (infant A, middle, end; infant B, middle, end; infant D, middle, end). Infant-directed speech (3rd tick) was seen in five segments (infant C, beginning, end; infant D, all segments). Variations between singing (2nd tick) and no vocalizations (lowest tick) were seen in few segments (infant A, middle, end; infant B, middle, end). Variations between no vocalizations (lowest tick) and adult speech (highest tick) were seen in a single segment (infant C, end). Variations between infant-directed speech (3rd tick) and adult speech (highest tick) were seen in infant C, end. Variations between infant-directed speech (3rd tick) and no vocalizations (lowest tick) were seen in infant D, beginning.

In summary, variations in infants’ behaviors occurred rarely, which was expected given that they were asleep or drowsy. More variations were seen in the mothers’ behaviors. Frequent variations in maternal affect and head orientation occurred during singing (mother A, segments 2 and 3, Figure 1).

#### 3.1.3. Frame-by-Frame Micro Scales

The frame-by-frame scales were coded for dyad D as part of training only and are shown in Figure 2.

### 3.2. Inter-Rater Reliability

Our findings show behaviors that can be coded reliably (reaching high inter-rater agreements): all macro scales; infant eye-opening, hands, and head orientation; and maternal head orientation and vocalizations. The three behaviors coded in macro scales are shown in Table 3. Raters agreed on most (23/27) of the coded macro behaviors. There was full agreement on infant body orientation. For infant states of alertness, agreement occurred in 8/9 [89%]; for maternal affective tone, it occurred in 6/9 [67%] of all videos. Any divergent ratings are specified in Table 3.

Inter-rater reliability results for the six behaviors coded in 1 μs scales are shown in Table 3. High agreement (kappa ≥ 0.90) was achieved for four behaviors (infant eyes, infant head orientation, maternal head orientation, and maternal vocalizations). Moderate reliability (kappa ≥ 0.70) was achieved for two behaviors (infant hand position; infant states of alertness).

### 3.3. Contingent Behaviors During IDS (Table 4)

A significant association was found between maternal IDS and several infant behaviors. Specifically, infants were significantly less likely to open their eyes when the mother was singing (*p* < 0.001), and significantly more likely to turn their head toward the mother (*p* < 0.001). There was no significant association between maternal singing and infant hand centering (*p* = 0.85). When examining maternal head orientation, infants were significantly less likely to open their eyes when the mother’s head was oriented toward them (*p* < 0.001), and less likely to have their hands centered (*p* < 0.001). Infants were also significantly less likely to turn their heads toward the mother when she was turning her head toward them (*p* = < 0.001) (Table 4).

**Table 4:** Coordinated behaviors of infant and mother—all dyads combined.

Infant	Mother		OR (95% CI)	*p*
	Not Singing	Singing		
Eyes closed	745	416	0 (0 to 0.04)	<0.001
Eyes open	177	0		
Hands extended	22	13	0.91 (0.43 to 2.05)	0.85
Hands centered	256	139		
Head not toward mother	618	151	3.54 (2.76 to 4.55)	<0.001
Head toward mother	306	265		
	Head not toward infant	Head toward infant		
Eyes closed	341	826	0.11 (0.07 to 0.17)	<0.001
Eyes open	138	39		
Hands extended	5	30	0.22 (0.08 to 0.59)	<0.001
Hands centered	169	226		
Head not toward mother *	213	546	0.47 (0.37 to 0.59)	<0.001
Head toward mother	266	320		

Note. Displaying total number of seconds across all 12 videos of all 4 dyads. OR—odds ratio; CI—confidence interval. * Head away from mother or frontal.

## 4. Discussion

The current study aimed to explore the feasibility of conducting a quantitative microanalysis of contingency between preterm infants and their mothers’ behaviors, during supported IDS within the MT context. We demonstrated that contingent mother–infant behaviors can be measured during supported IDS. These interactions may, after further validation, be used in trials as indicators of mother–infant interactions important for neurodevelopmental outcomes. Our study identified feasible behavioral scales for evaluating contingency between preterm infants and their parents during IDS. It also demonstrated that maternal IDS is significantly linked to infant-regulated behaviors—specifically, more frequent head orientation toward the mother and less eye opening. These findings may have important implications for understanding how early mother–preterm infant interactions relate to long-term developmental outcomes.

In our study, we developed new behavioral scales to code preterm infant behaviors. Previous studies have proposed feasible scales for measuring contingent behaviors between parents and infants [18,50], using markers such as infant gaze and expression. However, our observations of infants during IDS revealed challenges in identifying distinct gaze behaviors and reflexive smiles, as described in Lavelli and colleagues’ work [18]. These challenges may have been due to the unique IDS context, which could have stimulated more relaxed responses rather than facilitating alert states and interactions.

A similar discrepancy was observed in coding maternal affect, a common marker for assessing parent–infant interactions in infancy. Our findings indicated that during IDS, maternal affect was predominantly negative or neutral, with low inter-rater agreement. We suspect this was due to the unique phenomenon of “aesthetic emotions” [60] that can occur during musical engagement such as singing. For example, a mother may experience bonding, connection, and even happiness while singing a lullaby, yet her affect may be coded as “negative” due to the nature of the observed behavior, leading to disagreement between raters and low inter-rater reliability.

To address this, we focused on other well-established behavioral markers for assessing maternal behaviors and prioritized detectable infant behaviors. Our findings highlight infant and maternal behaviors that are feasible to code during all sessions of IDS with this population and reached high inter-rater agreements. Specifically, infant eye opening, hands, and head orientation, and maternal head orientation and vocalizations demonstrated high inter-rater reliability, suggesting that these behaviors can be measured reliably in very young, very preterm infants during IDS.

Additionally, we introduce a new scale for coding the infant hand position, a behavioral marker not previously articulated or measured in mother–infant interaction studies. Infant hand positioning and head orientation serve as markers for infants’ state of regulation/dysregulation during interactions based on the guidelines of the neonatal individualized developmental care and assessment program (NIDCAP [61,62]). By developing a reliable tool to assess the quality of interaction at an early age, contingency between maternal and infant behaviors could be used as a marker associated with diagnostic-related variables of preterm infants, and a tool that may help in predicting long-term developmental outcomes.

Our overall description of behaviors in the dyads shows an observable process of relaxation in two cases where the infant was not relaxed in segments 1 and 2 and became relaxed in segment 3 (dyad D, dyad B). In all cases, during the last singing segment, the infant was asleep, showing no behavioral signs of distress. These findings align with previous IDS studies using HRV and other physiological measures, indicating relaxation during maternal IDS [29,63,64] and music listening in the NICU [65]. However, this feasibility trial did not aim to evaluate the effectiveness of IDS; therefore, further research needs to test this more directly by comparing IDS with standard care or infant-directed reading (IDR) of the same duration. Music interventions in the early stages in the NICU aim to support relaxation, quiet alert states, and sleep, which is critical for healthy brain development in preterm infants [66,67]. For this reason, the songs integrated into MT are lullabies and/or songs of kin sung in a lullaby manner, such as in the current study, and not play songs [28,55,68,69].

Our initial hypothesis was that synchrony and/or contingency would be feasible to calculate. However, the infants in our study were mostly asleep and showed a low frequency of variations in their behaviors, making it impossible to use time-lagged analyses [18,50,51]. We therefore measured contingency between behaviors. Three of our examined preterm infants were around 35 weeks of post-menstrual age before they were discharged. Of note is that the participating preterm infants were not all at the same post-menstrual age (PMA); however, we cannot conclude that differences in behaviors are related to their PMA due to the small sample size of this study. Our findings indicate that IDS is significantly associated with distinct infant behavioral responses. Infants were more likely to orient their heads toward the mother when she was singing, suggesting that maternal vocalization acts as a salient social cue that draws infant attention. This is in agreement with previous research showing preterm infants’ ability to detect contingency and coordinate their vocalizations to the temporal aspects of maternal IDS [47,48]. At the same time, infants were significantly less likely to open their eyes during maternal singing, potentially reflecting a regulatory response of relaxation, also aligning with previous studies on the sedative effects of IDS in the NICU [67] and studies on older, term-born infants’ different reactions to lullabies versus play songs [36]. Regarding other behaviors, our findings show that infants are less likely to have eyes open when the mother’s head is oriented toward them (*p*-value < 0.001), and less likely to have their hands centered when the mother’s head is oriented toward them (*p*-value < 0.001). This finding may indicate that maternal gaze was elicited by infants’ signals of distress; however, more markers are needed to determine signs of distress. Another interesting finding was that infants were less likely to turn their heads toward their mother when she was looking at them, which may imply that the mothers were looking at their infants after detecting that the infants were less relaxed or interactive (e.g., Figure 1: dyad C beginning—infant’s head is oriented to the front, and not toward the mother, while the mother’s head is oriented toward the infant). We found no significant association between whether the infants’ hands were centered and the mothers were singing. However, in half of the cases, the hands were covered and coding was unfeasible.

Our study shows that very and extremely preterm infants, who are considered an at-risk population, demonstrate contingency with their parents’ behaviors during IDS, even though IDS at this stage aims to support sleep and relaxed reactions, rather than alert states. This is in agreement with other studies showing contingent behaviors in preterm infants [18,47,48,50]. Early interpersonal coordination—as shown in behavioral, physiological, and neural contingency and synchrony—is foundational for infants’ neurodevelopment and has been linked to specific long-term outcomes, including language acquisition [36,37], the maturation of social cognition and emotional regulation [35,38], and adaptive stress responsiveness and attachment security [10,39]. Our findings cannot support or refute that early interventions, such as supported IDS, may facilitate better parent–preterm infant interactions and coordination; however, this needs to be further tested in larger-scale longitudinal, multi-session, controlled studies. To illustrate the potential sample sizes required in such studies, we conducted a power analysis (function pwrss.z.logreg from R package pwrss). Sample sizes needed to achieve 80% for the odds ratios observed as significant in Table 4 ranged from *n* = 27 observations (for OR = 0.11) to n = 115 observations (for OR = 0.47). Further details would depend on the specific study design.

Our findings do not clarify whether the observed behaviors reflect infant responsiveness, maternal attunement, or mutual co-regulation. We demonstrate in this feasibility study that preterm infants around 35 weeks’ gestation show behaviors of coordinated interactions, but a larger study in older infants while infants are in an awake state might trace the temporal order of maternal and infant behaviors during IDS, or experimental designs that manipulate maternal input [70]. The coding process is quite complex and time-consuming in our present design, but developing this coding and potentially automating it using machine-based analysis could make its use more feasible in large sample sizes. It may be possible to observe a contingency between the onset of maternal singing and a change in the infant’s alertness (or other behaviors indicating relaxation, such as closing eyes or centering hands). Other measures that can be used for studying interactions at these young ages are brain activities (hyperscanning) or other neurological biomarkers such as those employed in Nguyen and colleagues’ work [36], as well as physiological markers such as HRV, heart rate, and respiratory rate, which have worked well in previous studies [63,64].

### Limitations

This study has several limitations. First, the small sample size (four mother–infant dyads) limits the generalizability of the findings and calls for further research with a larger, more diverse population. Second, lack of high behavioral variations prevented us from performing the planned calculations of synchrony; however, we could still calculate contingency. Given that each dyad was observed in only one session, our results should be interpreted as reflecting a potentially momentary situation rather than enduring characteristics. However, as our micro-observations include many codes that coincide with one another, we assume that all of them point toward a common trend. Another limitation is that all infants were held in a cradle position instead of the usual skin-to-skin contact to ensure the visibility of their faces during filming. This deviation from their typical holding position may have influenced their natural behavioral responses. Furthermore, because the analyses were based on aggregated cross-sectional frequency counts, they cannot capture the real-time sequence of behavior that would clarify who is responding to whom.

## 5. Conclusions

Our study highlights feasible behavioral scales for assessing contingency in preterm infants and parents during IDS, and it demonstrates that maternal IDS is significantly associated with infants’ regulated behaviors, including an increased head orientation toward the mother and reduced eye opening. Our results may be important for examining associations between mother–preterm infant interactions and long-term neurodevelopmental outcomes.

## Figures and Tables

**Table 1:** Demographics of infants and mothers.

	Infant									Mother		
Subject ID	Sex	GA (Weeks; Days)	PMA at Study Entry (Weeks; Days)	Weight at Birth (Grams)	IVH	PVL	BPD	Duration of Oxygen Support (Days)	ROP	Age (Years)	Education (Years)	Parity
A	f	31; 3	35; 3	1730	Grade 1—right	No	No	11	None	41	15	2
B	m	31; 4	35; 2	1748	No	No	No	3	None	30	15	2
C	f	24; 2	36; 2	640	Grade 2—Left	No	No	42	Stage 1—Left eye	31	15	1
D	f	27; 6	38; 3	660	No	No	No	56	Stage 2—Left eye	27	12	1

f—female, m—male, GA—gestational age, PMA—post-menstrual age, IVH—intraventricular hemorrhage, PVL—periventricular leukomalacia, BPD—bronchopulmonary dysplasia, ROP—retinopathy of prematurity.

**Table 2:** Operational definitions of behaviors.

Behavior	Time Scale (Seconds)	Values	Description	Comments
Infant behaviors				
Body orientation	150	0—away from mother	body is oriented away from mother’s body	macro scale coding is according to the behavior that is observable for most of the video segment the head orientation is determined from the infant’s perspective
		1—frontal	body is oriented toward the ceiling	
		2—toward mother	body is oriented toward the mother	
Eye opening	1	0—eyes closed	eyes are closed	eyes can be either squeezed or relaxed. eyes could be very slightly open but the pupil is not seen
		1—eyes open	eyes are open	eyes can be either narrow or wide open with detectable pupil
Hand position	1	0—hands extended	palms extended or stretched facing away from infant	behavior may appear in any of the following manners: one or both hands are extended; fingers of one or two hands are spread (hand either flexed or extended); hands facing away from infant; hands flaccid (lay on the sides of the body); hands flail; airplane position; salute position
		1—hands centred	both hands are centered toward the infant	behavior may appear in any of the following manners: placed in front, close or touching the face; tucked close to body center or torso; one to mouth, one centred; clasped; one grasping (object), one centred
Head orientation	1	0—away from mother		the head orientation is determined from the infant’s perspective
		1—upwards (not used)	head faces up	
		2—downwards (not used)	head faces down	
		3—frontal	head faces the ceiling	
		4—towards mother		
States of alertness	150	0—fussy	distinct signs of discomfort: eye or mouth squinting, cry	
		1—quite alert	eyes are open and focused	
		2—drowsy	eyes open and close rapidly; eyes can be open but without focus	
		3—sleep	deep or light sleep state; eyes are closed with subtle to no bodily movements	movements can be either rapid or slow, as long as the infant is not fussy and eyes are closed
Movements	1/30 (only in dyad “y”)	0—no movement	no distinct or meaningful movement	movements are observable in facial organs (eyes, eyebrows, mouth, lips), head, or hands each movement is coded compared to the previous frame, unless it is a part of continuous movement within a continuous movement, a pause of up to half a second (15 frames) will be considered movement
		1—movement	big or distinct movement	
Maternal behaviors				
Affect	1	0—negative	negative emotions like distress, fretting, anger, sadness, or discontentment with mouth curled or grimacing	
		1—neutral	neither smile nor distress signs	
		2—positive	smile with the mouth (open or closed) turned upward (i.e., contraction of cheek muscle), possibly contraction of the under-eye muscle, causing wrinkles in the eye region	
Affective tone	150	0—negative	negative	macro scale determined according to the overall tone of the interaction from the parent side
		1—neutral	neutral	
		2—positive	positive	
Head orientation	1	0—away from infant	head faces ceiling, leans backwards, faces another adult	
		1—toward infant		
Vocalizations	1	0—no vocalization		
		1—speech	infant-directed speech, verbalizations, rhymes	
		2—singing	melodic vocalizations, humming	
		3—adult speech	speech with another adults present (another parent, researcher, staff member)	
Head movements	1/30 (only in dyad D)	0—no movement		each movement is coded compared to the previous frame, unless it is a part of continuous movement within a continuous movement, a pause of up to half a second (15 frames) will be considered movement
		1—movement		
Leg movements		0—no movement 1—movement		
Left hand movements		0—no movement 1—movement		
Right hand movements		0—no movement 1—movement		

Note. In all variables, NA—not visible/codable.

**Table 3:** Inter-rater reliability of coded behaviors (1 s).

Variable	N ^1^	Cohen’s Kappa (95% CI)
Infant body orientation	1056	NA ^2^
Infant eyes (I1)	1355	0.93 (0.90 to 0.96)
Infant hand position (I2)	426	0.79 (0.70 to 0.89)
Infant head orientation (I3)	1358	0.94 (0.93 to 0.96)
Infant states of alertness	1358	0.71 (0.69 to 0.77)
Maternal affect (M1)	1183	0.59 (0.55 to 0.63)
Maternal affective tone	1363	0.50 (0.45 to 0.55)
Maternal head orientation (M2)	1349	0.95 (0.93 to 0.97)
Maternal vocalizations (M3)	1342	0.95 (0.93 to 0.96)

Note. Reliability based on Cohen’s kappa, using time series from 9 video segments coded by 2 raters. ^1^ Number of valid data points (observations: seconds) across all 9 videos. ^2^ No variance, with complete agreement across raters.

## Data Availability

The original contributions presented in this study are included in the article/Supplementary Materials. Further inquiries can be directed to the corresponding author.

## Supplementary Materials

The following supporting information can be downloaded at: https://www.mdpi.com/article/10.3390/children12091273/s1, Table S1: Overview of video segments analyzed: Overall (macro) behaviors.

## Abbreviations

The following abbreviations are used in this manuscript:

NICU	Neonatal Intensive Care Unit
IDS	Infant Directed Singing
MT	Music Therapy
