# Supplementary material for: Mother–Preterm Infant Contingent Interactions During Supported Infant-Directed Singing in the NICU—A Feasibility Study
Source: Children (Basel). 2025 Sep 22;12(9):1273. doi: 10.3390/children12091273 (PMC12468415; doi:10.3390/children12091273)
Supplement: Supplementary file 1 [file children-12-01273-s001.zip › children-3818891-supplementary.pdf]

Table S1. Overview of video segments analyzed: Overall (macro) behaviors

Dyad	Segment	Infant behaviours		Maternal behaviours	
		Body orientation#	State of alertness#	Affective tone[1]	Comment
A	beginning	Frontal	sleep	neutral, negative	no singing (relaxation with ocean disc)
	middle	frontal	sleep	neutral	maternal singing
	end	frontal	sleep	neutral, negative	maternal singing
B	beginning	frontal	drowsy	neutral	no maternal singing (relaxation with ocean disc)
	middle	frontal	drowsy	neutral	some maternal singing
	end	frontal	sleep	neutral	some maternal singing
C	beginning	frontal	sleep	neutral, positive	no maternal singing (relaxation with ocean disc)
	middle	frontal	sleep, drowsy	neutral	no maternal singing (therapist sings)
	end	frontal	sleep	positive	no maternal singing (therapist sings)

D	beginning	frontal	drowsy	positive	no maternal singing (relaxation with ocean disc)
	middle	frontal	drowsy	positive	some maternal singing
	end	towards	sleep	positive	maternal singing

#Macro scale; agreement between 2 raters, except where 2 behaviours are listed indicating disagreement.
